# Clavien–Dindo classification for grading complications after total pharyngolaryngectomy and free jejunum transfer

**DOI:** 10.1371/journal.pone.0222570

**Published:** 2019-09-12

**Authors:** Shimpei Miyamoto, Junichi Nakao, Takuya Higashino, Seiichi Yoshimoto, Ryuichi Hayashi, Minoru Sakuraba

**Affiliations:** 1 Department of Plastic and Reconstructive Surgery, National Cancer Center Hospital, Tokyo, Japan; 2 Department of Plastic and Reconstructive Surgery, Nippon Medical School Hospital, Tokyo, Japan; 3 Department of Plastic and Reconstructive Surgery, National Cancer Center Hospital East, Kashiwa, Japan; 4 Department of Head and Neck Oncology, National Cancer Center Hospital, Tokyo, Japan; 5 Department of Head and Neck Surgery, National Cancer Center Hospital East, Kashiwa, Japan; 6 Department of Plastic and Reconstructive Surgery, Iwate Medical University, Morioka, Japan; University of Texas Health Science Center Houston, UNITED STATES

## Abstract

**Background:**

The frequency of postoperative complications is used as an indicator of surgical quality; however, comparison of outcomes is hampered by a lack of agreement on the definition of complications and their severity. A standard grading system for surgical complications is necessary to improve the quality of clinical research and reporting in head and neck reconstruction.

**Methods:**

The aim of this study was to compare postoperative morbidity after microvascular head and neck reconstruction between patients with versus without a history of prior radiation therapy (RT) by using the Clavien–Dindo classification. A group of 274 patients was divided into two cohorts based on the history of prior RT: the RT group included 79 patients and the non-RT group included 195 patients. Postoperative (30-day) complications were compared between the groups with a nonstandardized evaluation system and the Clavien–Dindo classification.

**Results:**

The grades of complications according to the Clavien–Dindo classification were significantly higher in the RT group than in the non-RT group. The frequency of postoperative complications did not differ significantly between the groups according to the nonstandardized evaluation system.

**Conclusions:**

The Clavien–Dindo classification could serve as a useful, highly objective tool for grading operative morbidity after microvascular head and neck reconstruction when comparing similar defects and methods of reconstruction. Widespread use of the Clavien–Dindo classification system would allow adequate comparisons of surgical outcomes among different surgeons, centers, and therapies.

## Introduction

Free flap surgery has become the standard method of head and neck reconstruction, and the outcomes of these procedures have been investigated by several authors. [1] The frequency of postoperative complications is used as an indicator of surgical quality; however, comparison of outcomes is hampered by a lack of agreement on the definition of complications and their severity. [2] Most reports have used a nonstandardized system for evaluating postoperative complications and have not taken the severity of complications into account. Terms such as “minor,” “moderate,” and “severe” have been used, but they are subjective, unreliable, and often defined differently by each author. [3] A standard grading system for surgical complications is necessary to improve the quality of clinical research and reporting in head and neck reconstruction. [4]

The Clavien–Dindo classification (CDC) is a standardized system for the registration of surgical complications. The classification was initially developed by Clavien in 1992 for reporting negative outcomes after cholecystectomy and was modified by Dindo et al. in 2004 to increase its accuracy and acceptability in clinical practice. [5, 6] The major characteristic of the CDC system is that the severity of a complication is graded based on the type of therapy required to treat the complication. The CDC system has been validated and accepted worldwide for use in many fields of surgery. [7–9] Adaptations of the CDC system for head and neck reconstruction have been reported by several authors; however, the system has not yet been widely used in head and neck reconstruction and its applicability in this field remains unclear.[1, 10, 11]

The main objective of this study was to compare postoperative complications after total pharyngolaryngectomy (TPL) and free jejunum transfer (FJT) in patients with versus without a history of prior radiation therapy (RT) by using the CDC system. We previously conducted a similar study using a nonstandardized system for evaluating postoperative complications and failed to find a significant correlation between prior RT and the risk of postoperative complications. [12] We hypothesized that adaptation of the CDC system would help elucidate the impact of prior RT on the incidence of postoperative complications in patients after TPL with FJT.

## Materials and methods

A total of 274 patients who underwent FJT after TPL for biopsy-proven squamous cell carcinoma at our center from April 2010 through August 2016 were enrolled in this retrospective study. The study cohort included 236 men and 38 women with an age range from 44 to 87 years. This retrospective study was approved by an institutional review board (2017–343).

The patients were divided into two groups based on a history of prior RT to the neck: 79 patients had received RT and 195 patients had not. Radiation dose ranged from 44 Gy to136 Gy; the median dose was 70 Gy. Among the 79 patients in the RT group, 52 (65.8%) had undergone concurrent chemoradiotherapy and the remaining 27 (34.2%) had undergone RT alone. For most patients in the RT group (65 of 79, 82.3%), the procedure was salvage surgery after failed RT. The remaining 14 patients (17.7%) had a history of RT for another neck disease and TPL was performed for a second primary cancer.

Medical records of the 274 patients were analyzed for the following variables: sex, age, preoperative body mass index, American Society of Anesthesiologists Physical Status (ASA-PS) classification, history of smoking, primary site of tumor requiring TPL, history of prior chemotherapy for the disease, and postoperative complications within 30 days after surgery. Postoperative complications analyzed included recipient-site complications, donor-site (abdominal) complications, and medical complications. The recipient-site complications analyzed included anastomotic thrombosis, loss of the jejunum, pharyngocutaneous fistula, surgical-site infection of the neck, tracheal stoma necrosis, lymphorrhea, and hematoma.

### Statistical analysis

The analysis of postoperative complications was performed with two methods. First, a conventional analysis was performed, which we defined as a nonstandardized evaluation system. The frequencies of the complications described above were calculated and compared between the RT and non-RT groups. This system was the same as that described in our previous study.[12] Second, the analysis was performed using the CDC system. The most severe postoperative complication for each patient was graded with the CDC system (Table 1).[6] The grade for each complication was defined according to the Japan Clinical Oncology Group postoperative complications (JCOG PC) criteria.[13] If there was no appropriate term for an adverse event in the JCOG PC criteria, we assessed the complication as “Others (NO AE term)” in the criteria. The Clavien–Dindo (CD) grades for postoperative complications were compared between the RT and non-RT groups. In addition, the CD grades for postoperative complications excluding abdominal complications were compared between the two groups.

**Table 1:** Clavien–Dindo classification of surgical complications^6^.

Grade	Definition
Grade I	Any deviation from the normal postoperative course without the need for pharmacological treatment or surgical, endoscopic, and radiological interventions Allowed therapeutic regimens are: drugs as antiemetics, antipyretics, analgetics, diuretics, electrolytes, and physiotherapy. This grade also includes wound infections opened at the bedside
Grade II	Requiring pharmacological treatment with drugs other than such allowed for grade I complications Blood transfusions and total parenteral nutrition are also included
Grade III	Requiring surgical, endoscopic or radiological intervention
IIIa	Intervention not under general anesthesia
IIIb	Intervention under general anesthesia
Grade IV	Life-threatening complication (including CNS complications)* requiring IC/ICU management
IVa	Single organ dysfunction (including dialysis)
IVb	Multiorgan dysfunction
Grade V	Death of a patient

*Brain hemorrhage, ischemic stroke, subarachnoid bleeding, but excluding transient ischemic attacks.

CNS, central nervous system; IC, intermediate care; ICU, intensive care unit.

Statistical analysis was performed with IBM SPSS Statistics for Windows 23 (IBM, Armonk, NY, USA). Quantitative variables were analyzed with Student’s t-test. Categorical variables were analyzed with the chi-squared test, Fisher’s exact test, or Mann–Whitney U test.

In addition, to investigate risk factors for complications ≥ grade III, variables were included in univariate analysis using Fisher’s exact test. Variables with a *p* value less than 0.20 in univariate analysis were then included in multivariate logistic regression analysis.

Differences with a *p* value of less than 0.05 were considered statistically significant.

## Results

The data of patients in the RT and non-RT groups are summarized in Table 2. Sex distribution and patient age did not differ significantly between the groups. ASA-PS grades did not differ significantly between the groups; however, the grades tended to be higher in the non-RT group than in the RT group. A history of prior chemotherapy was significantly more common in the RT group.

**Table 2:** Comparison of patient characteristics between RT and non-RT groups (n = 274).

	RT	Non-RT	P
Number of cases	79	195	
Sex			
Male	66	170	0.43*
Female	13	25	
Age in years, mean (range)	67.8 (51–87)	68.5 (44–87)	0.52†
Body mass index, kg/m^2^, mean (range)	19.8 (12.9–30.2)	20.6 (14.2–31.7)	0.06†
ASA-PS			
1	36	66	0.05 ‡
2	39	111	
3	4	18	
History of smoking+			
Yes	51	146	0.09*
No	28	49	
Primary site of tumor requiring TPL			
Hypopharynx	50	152	
Cervical esophagus	19	31	
Larynx	8	7	
Oropharynx	2	5	
Prior chemotherapy for the disease			
Yes	52	3	0.00††
No	27	194	

Data are numbers of patients unless otherwise indicated.

RT, radiation therapy; ASA-PS, American Society of Anesthesiologists-Physical Status; TPL, total pharyngolaryngectomy.

*Chi-squared test

† Student’s *t*-test

‡ Mann–Whitney U test

†† Fisher’s exact test

Comparisons of postoperative complications between the RT and non-RT groups using the nonstandardized evaluation system are shown in Table 3. The frequencies of recipient-site, donor-site (abdominal), and medical complications did not differ significantly between the groups. Furthermore, the frequency of each individual type of complication did not differ significantly between the RT and non-RT groups.

**Table 3:** Comparison of postoperative complications between RT and non-RT groups according to a nonstandardized evaluation system.

	No. of patients (%)		P*
Complication	RT (n = 79)	Non-RT (n = 195)	
Neck complications	25 (31.6)	49 (25.1)	0.29
Anastomotic thrombosis	3 (3.8)	5 (2.6)	0.69
Loss of the jejunum	3 (3.8)	3 (1.5)	0.36
PCF	4 (5.1)	3 (1.5)	0.11
SSI	11 (13.9)	15 (7.7)	0.12
Tracheal stoma necrosis	5 (6.3)	13 (6.7)	1
Lymphorrhea	2 (2.5)	10 (5.1)	0.52
Hematoma	3 (3.8)	5 (2.6)	0.69
Abdominal complications	10 (12.7)	20 (10.3)	0.67
Ileus	2 (2.5)	2 (1.0)	0.35
Dehiscence	5 (6.3)	4 (2.1)	0.13
SSI	3 (3.8)	15 (7.7)	0.29
Medical complications	8 (10.1)	11 (5.6)	0.20
Pneumonia	2 (2.5)	1 (0.5)	0.20
Supraventricular arrhythmia	0	3 (1.5)	0.56
Heart failure	1 (1.3)	1 (0.5)	0.49
Renal failure	0	1 (0.5)	0.52
Sepsis	2 (2.5)	0	0.08
Portal vein thrombosis	0	1 (0.5)	0.52
Anemia	1 (1.3)	4 (2.1)	1
Cholecystitis	1 (1.3)	0	0.29
Electrolyte imbalance	1 (1.3)	3 (1.5)	1

PCF, pharyngocutaneous fistula; RT, radiation therapy; SSI, surgical-site infection.

* Fisher’s exact test.

The CD grades for most postoperative complications could be defined with the JCOG PC criteria because we could find an appropriate adverse event term for each complication. However, “anastomotic thrombosis,” “loss of the jejunum,” “pharyngocutaneous fistula,” and “tracheal stoma necrosis” did not fit into any adverse event term in the JCOG PC. We classified these complications as “Others (No AE term)” in the criteria. Correlations between these complications and their CD grades are shown in Table 4.

**Table 4:** Correlation between specific head and neck complications and their grades according to Clavien–Dindo classification.

	No. of patients (No. in RT group, No. in Non-RT group)			
Complication	Clavien–Dindo classification (grade)			
	I	II	IIIa	IIIb
Anastomotic thrombosis	0	0	0	8 (3, 5)
Loss of the jejunum	0	0	0	6 (3, 3)
PCF	0	0	5 (4, 1)	2 (0, 2)
Tracheal stoma necrosis	11 (2, 9)	3 (1, 2)	1 (1, 0)	3 (1, 2)

PCF, pharyngocutaneous fistula; RT, radiation therapy.

Comparisons of postoperative complications between the RT and non-RT groups according to the CDC system are shown in Table 5. There were no deaths within 30 days (CD grade V), and the overall mortality was 0%. The CD grades for postoperative complications were significantly higher in the RT group than in the non-RT group. The CD grades for postoperative complications excluding abdominal complications were also significantly higher in the RT group than in the non-RT group.

**Table 5:** Comparison of postoperative complications between non-RT and RT groups according to Clavien–Dindo classification.

	All complications			Excluding abdominal complications		
Clavien–Dindo classification (grade)	No. of patients		P*	No. of patients		P*
	RT (n = 79)	Non-RT (n = 195)		RT (n = 79)	Non-RT (n = 195)	
(-)	41	124	0.02	46	136	0.04
I	12	34		11	26	
II	7	13		7	13	
IIIa	8	11		7	8	
IIIb	8	11		5	10	
IVa	1	1		1	1	
IVb	2	1		2	1	
V	0	0		0	0	

Left columns: grades including all complications

Right columns: grades excluding abdominal complications

RT, radiation therapy.

* Mann–Whitney U test

A total of 43 patients (15.7%) had complications ≥ grade III. Univariate analysis revealed that the factors significantly associated with an increased risk of complications ≥ grade III were advanced age (≥ 75 years) and prior RT (Table 6). Multivariate logistic regression analysis of these factors and body mass index showed that prior RT was an independent risk factor for complications ≥ grade III. The odds of a complication ≥ grade III among patients with prior RT was 2.5 times that among patients without prior RT (Table 7).

**Table 6:** Results of univariate analysis to assess risk factors for complications ≥ grade III (n = 274).

Variable	Total number (%)	Complications ≥ grade III, number (%)	P*
Age (years)			
<74 (non-advanced)	211 (77.0)	26 (12.3)	0.01
≥75 (advanced)	63 (23.0)	17 (27.0)	
Sex			
male	236 (86.1)	38 (16.1)	0.81
female	38 (13.9)	5 (13.2)	
Body mass index (kg/m^2^)			
<18.5 (underweight)	74 (27.0)	8 (10.8)	0.20
≥18.5 (not underweight)	200 (73.0)	35 (17.5)	
ASA-PS			
1 or 2	252 (92.0)	38 (15.1)	0.36
3	22 (8.0)	5 (22.7)	
History of smoking			
Yes	197 (71.9)	28 (14.2)	0.27
No	77 (28.1)	15 (19.5)	
Prior RT			
Yes	79 (28.8)	19 (24.0)	0.03
No	195 (71.2)	24 (12.3)	
Prior chemotherapy			
Yes	55 (20.1)	12 (21.8)	0.21
No	219 (79.9)	31 (14.2)	

ASA-PS, American Society of Anesthesiologists-Physical Status; RT, radiation therapy.

* Fisher’s exact test.

**Table 7:** Results of multivariate logistic regression analysis to assess risk factors for complications ≥ grade III (n = 274).

Variables	Odds ratio	95% confidence interval	P
Age (years)	1.036	0.993–1.082	0.10
Body mass index (kg/m^2^)	1.068	0.964–1.183	0.21
Prior RT	2.469	1.245–4.897	0.01

RT, radiation therapy.

## Discussion

This study demonstrated that the CD grades for postoperative complications were significantly higher in the RT group than in the non-RT group after TPL and FJT. However, a comparison of complications between the groups according to the nonstandardized evaluation system did not reveal statistically significant differences in any variable.

Several recent studies have adopted the CDC system for grading complications after major head and neck surgery. [1, 10, 11] Perisanidis et al. applied the CDC system to postoperative complications after free flap surgery for head and neck reconstruction in 79 patients who underwent FJT for oral and oropharyngeal defects.[1] They stated that the use of this system can ensure more consistency and a better quality of reporting in head and neck journals. McMahon et al. analyzed postoperative complications of 192 consecutive patients who underwent microvascular head and neck reconstruction.[11] They investigated predictors of major complications, defined as CD grade III or above. Nobel et al. reported a retrospective analysis of 304 consecutive head and neck reconstruction procedures, in which complications were classified with the CDC system.[10] They concluded that increased tumor stage and pharyngoesophageal reconstruction were statistically significant predictors of complication grades. The results of these studies confirmed the usefulness of the CDC system in clinical practice, as well as for reporting complications in research studies. However, the applicability of the CDC system in this patient population has yet to be established because previous studies have lacked comparative data from a nonstandardized evaluation system.[1, 10, 11]

In our study, the CDC system identified differences between the non-RT and RT groups, whereas the nonstandardized evaluation system failed to do so. The frequencies of each complication did not differ significantly between the groups, and this finding is consistent with that of our previous report.[12] These results suggest that grading of postoperative complications with a standardized system is important to evaluate the influence of prior RT on outcomes. Some previous studies have shown that prior RT increases flap failure and fistula formation in microvascular head and neck reconstruction; however, other studies could not confirm prior RT as a risk factor for operative morbidity. [14–16] A potential cause of these mixed results is that comparisons in previous reports were based only on the frequency of the complications and did not take severity into account. TPL with FJT is an established procedure with a low frequency of wound complications, even among patients with prior RT.[12] These low frequencies can make the detection of differences difficult. Wound complications in patients with prior RT may tend to be severe because of poor wound-healing potential.[17] Comparing non-RT and RT groups without grading the complications can therefore underestimate the severity of complications in the RT group, thus favoring the RT group.

One advantage of the CDC system is that the severity of complications is graded with high objectivity. Grades are assigned based on the type of treatment required to treat the complication. This approach avoids subjectivity and imprecision in reporting complications and eliminates the use of qualitative terms such as “mild,” “moderate,” and “severe” in grading complications. [10] Although the CDC system is not yet regularly used in the head and neck literature, the system would provide a common language among head and neck surgeons. If the CDC system is widely implemented for this purpose, adequate comparisons of surgical outcomes can be made among different surgeons, centers, and therapies.[6]

We acknowledge that there are several disadvantages of the CDC system. First, the grading of complications with the CDC system can be difficult in some cases and is subject to bias by the grader’s interpretation because only general grading criteria are defined originally.[13] Monteiro et al. reported that the interobserver reliability of the CDC system ranged from moderate to high when used to evaluate complications after head and neck surgery; however, the score tended to be lower in case scenarios that were subject to rater’s interpretation.[4] Although we did not investigate interobserver reliability in the current study, we attempted to eliminate this bias by using the JCOG PC criteria.[13] The JCOG PC criteria were established as supplementary criteria for the CDC system to standardize the terms used to define adverse events. These criteria specify commonly experienced surgical adverse events and provide more detailed grading guidelines based on the CDC system.

Second, the CDC system cannot distinguish between recipient-site complications and donor-site complications. Theoretically, there should be no relationship between prior RT to the neck and abdominal complications; however, the influence of abdominal complications on the results could not be excluded when the CDC grade for each patient was based on all complications. Modifications that can distinguish between recipient-site and donor-site complications may be more beneficial from a reconstructive surgeons’ perspective. In the current study, we performed subset analysis of the CDC grade without abdominal complications and obtained similar results to those that included all complications; however, the distinction was sometimes difficult. For example, a patient in the non-RT group in our study developed acute renal failure after ileus and was graded as CD grade IVa. Ileus-induced dehydration was speculated to be the main cause of renal failure; however, renal failure cannot be categorized as a donor-site complication because the etiology is usually multifactorial.

Third, it is possible that the CDC system cannot appropriately grade specific wound complications after microvascular head and neck surgery. In the current study, the incidence of “loss of the jejunum” and “pharyngocutaneous fistula” may have been underestimated compared with other wound complications. The occurrence of these complications could have a great impact on the postoperative course, including multiple take-backs, prolonged length of hospital stay, and the potential for catastrophic complications. However, these complications would be assigned the same grade as common wound complications such as a neck hematoma requiring drainage. [4] Further studies are warranted to modify the CDC system to differentiate these specific complications from other common complications and to better fit head and neck patients. Until that time, frequencies of these specific complications should be reported and analyzed simultaneously with the CDC grades.

The main limitation of this study is that this was a retrospective study and there is the possibility of underreporting of complications because of a lack of information. However, it has been noted that the use of the CDC system can prevent down-rating of major negative outcomes even in retrospective cohort studies.[6] The grading was determined on the basis of treatments, which is usually recorded in the patient’s medical chart.[18] The second limitation is that FJT is no longer the first choice for TPL reconstruction in some high-volume centers.[19–21] In some centers the anterolateral thigh flap (ALT) is the first choice; the reported frequency of wound complications does not differ significantly between FJT and ALT. However, these comparisons were made on the basis of a nonstandardized system and the severity of complications was not considered.[19] In addition, evaluation of donor-site complications becomes critical when evaluating the best donor site for TPL reconstruction. The CDC system, therefore, is suitable for this comparison because it can simultaneously evaluate the severity of recipient-site and donor-site complications. We await a further study using the CDC system to compare the severity of complications after TPL reconstruction with ALT versus FJT.

## Conclusion

The adaptation of the CDC system revealed a significant difference in the severity of postoperative complications between RT and non-RT groups after TPL and FJT. The CDC system could serve as a useful, highly objective tool for grading operative morbidity after head and neck reconstruction when comparing similar defects and methods of reconstruction. Widespread use of the CDC system would provide a common language among head and neck surgeons.

## Supporting information

S1 File Summary of the patients’ variables. (XLSX)
